# Genome-wide identification and functional characterization of *GPCR* family genes reveal their key roles in the vitellarium development and egg production in *Schistosoma japonicum*

**DOI:** 10.1186/s13071-025-06929-2

**Published:** 2025-07-17

**Authors:** Xiaoxu Wang, Chuantao Fang, Guofeng Cheng

**Affiliations:** 1 School of Biotechnology Jiangsu, University of Science and Technology, Zhen Jiang, 212100 China; 2 Shanghai Tenth People’s Hospital, Institute for Infectious Diseases and Vaccine Development, Tongji University School of Medicine, Shanghai, 200331 China; 3 Clinical Center for Brain and Spinal Cord Research, Tongji University, Shanghai, 200092 China; 4 Affiliated Shanghai Blue Cross Brain Hospital, School of Medicine, Tongji University, Shanghai, 200020 China

## Abstract

**Background:**

Schistosomiasis is a zoonotic parasitic disease of the genus *Schistosoma*. Current therapeutic approaches predominantly rely on the single drug praziquantel, highlighting the urgent need to identify additional effective drug targets. G protein-coupled receptors (GPCRs) play important roles in various biological processes and are considered pivotal in drug development. However, the current understanding of the function of GPCRs in schistosomes remains limited.

**Methods:**

We conducted a systematic bioinformatics analysis of *Schistosoma japonicum GPCR*s using public genomic resources to elucidate their molecular evolution and expression profiles. Selected *GPCR*s were functionally characterized using whole-mount in situ hybridization, double fluorescence in situ hybridization, and RNA interference.

**Results:**

Bioinformatics analysis identified 126 *GPCR* genes in *S. japonicum* and 8 *GPCR*s were selected for further studies. qPCR analyses revealed that EWB00_004787 (*Sj-Smo*) and EWB00_003955 (*Sj-imGPCR*) were significantly enriched in the vitellarium of female worms, where they were found to colocalize with *Sj-DDR48*. Following RNAi inhibition of *Sj-Smo* and *Sj-imGPCR*, cell proliferation in the vitellarium was significantly reduced by 73% and 54%, respectively, and egg productions were also markedly decreased.

**Conclusions:**

This study identifies *Sj-Smo* and *Sj-imGPCR* as essential regulators of vitellarium development and egg production in *S. japonicum*. Targeting these *GPCR*s may represent a potentially promising strategy to disrupt egg-mediated pathology and transmission for schistosomiasis control.

**Graphical Abstract:**

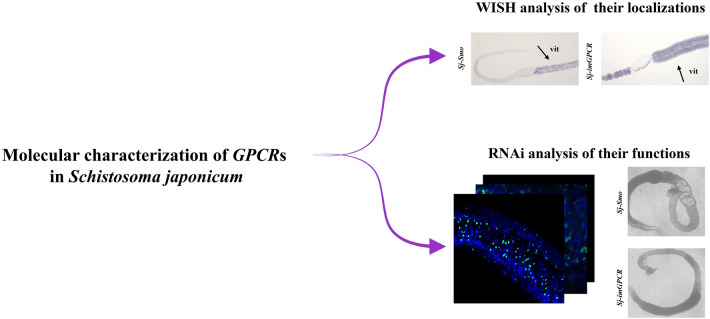

**Supplementary information:**

The online version contains supplementary material available at 10.1186/s13071-025-06929-2.

## Background

Schistosomiasis, a neglected tropical zoonosis, poses a significant threat to public health. According to data from the World Health Organization, schistosomiasis is endemic to 78 countries, with at least 251.4 million people infected [1, 2] and approximately 24,000 deaths worldwide as of the end of 2016 [3]. Adult schistosomes reside in the mesenteric veins of definitive hosts, mate, and lay a large number of eggs that are deposited in the liver and intestines, causing pathological reactions [4]. Therefore, deciphering the molecular mechanisms governing egg production and developing targeted interventions to disrupt this process, represents a critical strategy for schistosomiasis control, as eggs drive both pathology and transmission.

G protein-coupled receptors (GPCRs) are the largest family of receptors in the human genome, accounting for approximately 30% of drug targets [5]. They interact with diverse endogenous ligands, including small molecules, peptides, and proteins and modulate almost all aspects of human physiological functions [6]. In insects, GPCRs play an important role and are considered potential targets for insecticide development. GPCRs perform key functions in physiological processes, toxicological responses, and development of insecticide resistance in insects [7]. GPCRs are involved in regulating many biological processes [8]. A recent study on *S. japonicum* indicated that an allatostatin-A receptor-like gene encoding a typical GPCR may be involved in the regulation of reproductive development [9]. Next, we systematically analyzed GPCRs in *Schistosoma mansoni,* which indicated that they may not only contribute to gonad-specific functions but also to non-gonad pairing-dependent processes [8]. In *S. mansoni*, the rhodopsin orphan GPCR20 interacts with neuropeptides, including SmNPP26 and SmNPP40, affecting parasite growth, sexual differentiation, and egg production [10]. Moreover, studies on rhodopsin GPCRs in *S. mansoni* have indicated that some of them have high sequence similarity to the GPCRs from *Biomphalaria glabrata*, suggesting their potentially important roles in parasite–host interactions [11].

Although the aforementioned studies have demonstrated the important roles of GPCRs in *S. mansoni*, related studies on *S. japonicum* are limited. In the present study, we performed systematic analyses to identify the GPCRs of *S. japonicum* at the genome-wide level and then evaluated the functions of selected GPCRs in adult parasites using RNA interferences.

## Methods

### Animals and *S. japonicum* collection

The animal experiments were approved by the Shanghai Laboratory Animal Management Committee and Internal Review Panel of Laboratory Animal Research Center, Tongji University (TJAA00822501). A total of 30 healthy, Kunming mice (6 weeks of age) were purchased from Shanghai Jetset Laboratory Animals (Shanghai, China). Animal infections were performed as described previously [12, 13]. Each mouse was infected with approximately 120 *S. japonicum* cercariae strains (Jiangsu isolate; China). At 26 d postinfection, the mice were euthanized and adult worms were collected via hepatic portal perfusion. After washing with phosphate buffered saline (PBS), the worms were then transferred to RPMI 1640 medium (Adamas, China) containing 1% penicillin–streptomycin (Thermo Scientific, USA) and incubated for 4 h, after which the medium was replaced with AB169 medium [14].

### Bioinformatics analysis of *GPCRs* in *S. japonicum*

The transcriptome data of *S. japonicum* at different developmental time points were downloaded from NCBI SRA (https://www.ncbi.nlm.nih.gov/sra). The accession numbers and sample information are listed the follows: 14 d (SRR4267990, SRR4289346, SRR4292306), 16 d (SRR4289349, SRR4296931, SRR4279491), 18 d (SRR4289351, SRR4296934, SRR4279833), 20 d (SRR4289353, SRR4296936, SRR4279841), 22 d (SRR4289333, SRR4292206, SRR4296938), 24 d (SRR4289339, SRR4292292, SRR4296940), and 26 d (SRR4289341, SRR4292299, SRR4296942). The GPCRpred method (https://gpcr.utep.edu/) was used to classify the homologs of GPCRs in *S. japonicum* and predict their upstream ligands [15].

The transcript levels of *GPCR*s at different developmental stages in *S. japonicum* were analyzed using Mfuzz in R studio (4.2.2) and a heatmap was plotted using Complex Heatmap (version 3.10). The homology alignment analyses were conducted for different species, utilizing their respective genomes (*Mus musculus* (GRCm39), *Caenorhabditis elegans* (WBcel235), *Schistosoma haematobium* (GCA_000699445.3), *Schistosoma intercalatum* (GCA_944470365.2), *Schistosoma mansoni* (GCA_000237925.5), *S. japonicum* (GCA_006368765.1), and *Homo sapiens* (9606)). GPCR pathway analysis was performed using the Kyoto Encyclopedia of Genes and Genomes (KEGG) database. Subsequently, the putative protein–protein interactions (PPI) of the selected GPCRs were analyzed using the STRING database (https://string-db.org/), and a network diagram of potential PPI was constructed.

### Amplification of full-length coding DNA sequence (CDS) for *GPCRs* using PCR

Total RNA was extracted from parasites collected at 26 d postinfection (dpi) using TRIzol Reagent (Thermo Scientific, USA) following the manufacturer’s protocol. Subsequently, cDNA was synthesized using the Primescript RT Master Kit (TaKaRa, China). The reactions contained 5 μL of 5 × PrimeScript RT Master Mix and 300 ng total RNA, and nuclease-free H_2_O was added to bring the final volume to 10 μL. The mixture was incubated at 37 °C for 15 min, followed by that at 85 °C for 5 s. PCR was performed using the 2 × Hieff PCR Master Mix (Yeasen, China) and primers (Additional file 4: Table S1). The PCR reaction mixture contained 25 μL 2 × PCR Master Mix, 1 μL each of 10 μM forward and reverse primers, and 1 μL of cDNA template. Nuclease-free water was added to bring the final volume to 50 μL. The mixture was incubated at 95 °C for 5 min, followed by 35 cycles of 95 °C for 15 s, 55 °C for 15 s, and 72 °C for 30 s, and a final extension at 72 °C for 5 min. The PCR products were analyzed on 1.5% agarose gel and then submitted for sequencing (Sangon, China).

### qPCR analysis of the transcript levels of *GPCRs* in *S. japonicum*

Adult males and females (26 dpi) were isolated and subjected to RNA isolation. Adult females (26 dpi) were dissected under a microscope into three parts: the anterior, ovary, and vitellarium. Total RNA was extracted from each part using the TRIzol reagent (Thermo Scientific, USA). The RNA quality and integrity were analyzed via 1% agarose gel electrophoresis. cDNA was synthesized using a Primescript RT Master Kit (TaKaRa, China). The transcript levels of *GPCR*s in different plan parts were analyzed using qPCR combined with the listed primers (Jie Li Biological Company, China; Additional file 4: Table S1).

The efficiency of primer amplification for each gene was accessed using a standard curve. Briefly, cDNA templates were serially diluted fivefold (5⁻^1^ to 5⁻^5^) for qPCR analyses. A standard curve was generated by plotting the logarithm of the dilution factors against the corresponding Ct values. Primer efficiencies were calculated using the formula: Efficiency = 10^(–1/slope)−1^[16, 17].

qPCR was conducted using an ABI StepOne Plus Real-Time PCR System (Applied Biosystems, USA). The reaction solutions were composed of 10 μL ChamQ Universal SYBR qPCR Master Mix (Vazyme, China), 0.4 μL each of 10 μM forward and reverse primers, 1 μL cDNA template, and nuclease-free H_2_O was added to bring the final volume to 20 μL. The mixtures were incubated at 95 °C for 30 s, followed by 40 cycles of 95 °C for 10 s, and 60 °C for 30 s. Three replicates were performed for each sample. *S. japonicum NADH* [18, 19] was used as an internal control.

### Whole-mount in situ hybridization

Primers were designed by introducing the T7 promoter sequence into the selected *GPCR*s (EWB00_004787 and EWB00_003955) (Additional File 4: Table S1). The PCR components, except for the primers and reaction condition, were the same as those described above. PCR products were purified using the GeneJET PCR Purification Kit (Thermo Scientific, USA). DIG-labelled probes were prepared using the MEGAscript kit (Thermo Scientific, USA). Briefly, the reaction mixture contained 100 ng PCR product, with 2 μL each of ATP, CTP, GTP, and UTP solution, 2 μL 10 × T7 buffer, 2 μL T7 RNA polymerase, 0.7 μL Digoxigenin 11-UTP (Roche, Switzerland), and nuclease-free H_2_O was added to bring the final volume to 20 μL. The mixtures were incubated at 37 °C for 8 h, and 1 μL DNase I (Thermo Scientific, USA) was added and then incubated at 37 °C for 15 min. Regarding probe purification, 30 μL of nuclease-free H_2_O and 30 μL of lithium chloride precipitation solution were added to the above reaction solution. The mixtures were incubated at −20 °C for 1 h. Next, the solutions were centrifuged at 4 °C for 15 min at maximum speed. The precipitates were further washed with 75% ethanol, dried at room temperature under airflow, and then dissolved into nuclease-free H_2_O. The probe concentration was determined using a NanoDrop spectrophotometer (Thermo Scientific, USA).

Whole-mount in situ hybridization (WISH) experiments were conducted as previously described [20]. Briefly, parasites were collected from infected animals at 26 dpi, and males and females were manually separated. Female parasites were used for the WISH experiment. The females were washed twice with PBSTx (0.3% Triton X-100 (Merck KGaA, Germany) in PBS) and then treated with 0.6 M MgCl_2_ for 1 min. Upon the fixation of parasites in 4% formaldehyde PBSTx solution for 4 h, the worms were dehydrated in 100% methanol at −20 °C overnight. Next, the worms were rehydrated in a graded series of methanol solutions (100%, 50%) and then treated in bleaching solution (9 mL nuclease-free H_2_O, 500 μL formamide (Merck, Germany), 250 μL 20 × SSC (Thermo Scientific, USA), and 400 μL 30% H_2_O_2_) under light for 3 h. Thereafter, the parasites were treated with 5 μg/mL Proteinase K (Thermo Scientific, USA) for 45 min and further fixed with 4% formaldehyde solution for 10 min. Prehybridization was performed at 52 °C for 2 h. For hybridization, the prepared probe (100 ng/mL) was added to the hybridization solution (50% De-ionized Formamide (Roche, Switzerland), 10% Dextran Sulfate (Merck KGaA, Germany), 5 × SSC, 1 mg/ml yeast RNA (Merck, Germany), 1% Tween-20), and then incubated for 16 h at 52 °C in hybridization oven (Gene Company, China). Next, the worms were treated with Colorimetric Block Solution (7.5% horse serum in TNTx, Thermo Scientific, USA) at room temperature for 2 h and then incubated with Anti-Digoxigenin-AP antibody (1:2000 dilution) (Roche, Switzerland) overnight at 4 °C. Next, the worms were rinsed with TNTx and developed in BCIP/NBT substrate (Roche, Switzerland) prepared with AP buffer (0.1 M Tris, PH 9.5; 0.1 M NaCl in ddH_2_O). After development, the parasites were decolorized in 100% ethanol and transferred to 80% glycerol (Greagent, China) for observation under a microscopy (Motic, China).

### Double fluorescence in situ hybridization (FISH) experiment

FISH was performed. Briefly, PCR was used to amplify the fragments of the target genes (EWB00_004787, EWB00_003955 and EWB00_005782) (Additional file 4: Table S1). Upon the purification of PCR products, the in vitro transcription reaction system was prepared according to the MEGAscript kit protocol: 100 ng of purified PCR product was mixed with 2 μL each of ATP, CTP, GTP, and UTP solution, 2 μL of 10 × T7 transcription buffer, 2 μL of T7 RNA polymerase, and 0.7 μL of digoxigenin-11-UTP (for DIG-labeled probes) or FITC-12-UTP (for FITC-labeled probes) (Roche, Switzerland), with nuclease-free water being added to reach a final volume of 20 μL, where *Sj-DDR48* and *Sj-imGPCR* were labeled with FITC, while *Sj-Smo* and *Sj-imGPCR* were labeled with DIG. The reaction mixtures were incubated at 37 °C for 8 h. Thereafter, 1 μL of DNase I was added to degrade the DNA template and then incubated at 37 °C for 15 min. RNA purification was performed as described for WISH probe preparation. The probe concentration was determined using a NanoDrop spectrophotometer (Thermo Scientific, USA).

Double-fluorescence in situ hybridization experiments were performed as previously described [10]. After hybridization, the worms were blocked with Colorimetric Block Solution (5% horse serum) (Thermo Scientific, USA), 0.5% Roche Western blocking (Roche, Switzerland) for 2 h at room temperature, and then incubated with the antibody (1:2000 dilutions, Anti-Digoxigenin-POD in Block solution, Roche, Switzerland) overnight at 4 °C. After blocking, the worms were treated with TNTx. Thereafter, Cy3 TSA solution (5 μL Cy3 in 500 μL 1 × Amplification Diluent; TSA Fluorescence System Kit, YEASEN, China) was added and incubated for 10 min and then treated with 3% H_2_O_2_ for 1 h at room temperature. Next, the worms were incubated with the antibody (1:2000 dilutions, Anti-Fluorescein-POD in Block solution, Roche, Switzerland) overnight at 4 °C. Then, Cy5 TSA solution (5 μL Cy5 was added in 500 μL 1 × Amplification Diluent; TSA Fluorescence System Kit, YEASEN, China) was added and incubated for 10 min. The parasites were transferred to 80% glycerol, incubated for 1 h, and mounted. The parasites were observed via confocal microscopy (ZEISS, Germany) at excitation wavelengths of 550 nm and 648 nm [21].

### RNA interference procedure

Double-stranded RNA (dsRNA) was prepared as previously described [22]. Target genes (EWB00_004787 and EWB00_003955) were amplified via PCR using specific primers (Additional file 4: Table S1). dsRNAs was synthesized using a MAXIscript kit (Thermo Scientific, USA). The reactions mixture composed: 1 μg PCR product; 8 μL ATP, CTP, GTP, and UTP solution; 2 μL 10 × T7 buffer; 2 μL T7 RNA polymerase, and nuclease-free H_2_O added to make the final volume 20 μL. The reaction mixtures were incubated at 37 °C for 8 h, followed by the addition of 1 μL of DNase (Thermo Scientific, USA), and incubated at 37 °C for 15 min. Then, the mixtures underwent gradient annealing at 95 °C for 3 min, 75 °C for 3 min, and 55 °C for 3 min. The annealed product was analyzed via electrophoresis (Bio-Rad, USA).

Worms were collected at 26 dpi, and females were cultured in 12-well plates with 10 worms per well (three biological replicates). dsRNA (30 μg/mL) for *Sj-Smo* or *Sj-imGPCR* was added on the cultured wells. The control groups were treated with an unrelated dsRNA. Worms were cultured for 12 d. During the culture period, the number of eggs was counted every 2 d when the culture medium was changed to track changes in egg production. At 12 d posttreatment, worms were collected for qPCR analysis to determine the altered levels of target genes at the transcript level. Worm morphology was observed under a microscope (Motic, China).

### FastBlue BB staining and EdU staining

FastBlue BB staining was performed as previously described [14]. Female worms were fixed for 4 h with 4% formaldehyde in PBSTx and then incubated with 10 mg/mL FastBlue BB (dissolved in PBSTx, Thermo Scientific, USA) for 5 min at room temperature. After development, the samples were rinsed with PBSTx for three times. Morphological changes in the female reproductive system were assessed, the parasites were cleared using 80% glycerol and then mounted on slides for microscopical observation.

The BeyoClick™ EdU-488 Cell Proliferation Detection Kit (Beyotime, China) was used for EdU staining. Female worms were treated with 10 μM EdU for 21 h, then collected, and incubated with 0.6 M MgCl_2_ for 1 min. Next, the worms were fixed with 4% formaldehyde in PBSTx for 4 h, followed by dehydration in methanol. Next, the worms were rehydrated in 50% methanol in PBSTx, bleached under light for 3 h, and then treated with 5 μg/mL Proteinase K (Thermo Scientific, USA) in 1 × PBSTx for 45 min. The parasites were further post-fixed with 4% formaldehyde in PBSTx for 10 min. Subsequently, the parasites were incubated in the EdU detection solution (430 μL click reaction buffer, 10 μL 100 mM CuSO_4_, 1 μL 10 mM Azide-fluor 488, 50 μL Click additive solution) in the dark for 30 min. Then, the worms were transferred into DAPI solution (1 μg/mL in PBSTx, Beyotime, China) for 1 h incubation. The worms were further cleared with 80% glycerol and mounted using Vectashield (CITOTEST, China). EdU-stained worms were observed using a confocal microscope. The worms were adjusted to the optimal angle in a single Z-plane, with a particular focus on changes in the vitellarium. DAPI was excited using a 340 nm laser, and Alexa fluor 488 was excited using a 488 nm argon ion laser (ZEISS, Germany).

Then, ImageJ2 (https://imagej.net/ij/) was used to assess cell proliferation [23]. EdU-positive cells were segmented from the background using thresholding, and the number of EdU-positive cells was automatically identified and counted using the Analyze Particles function in ImageJ2, with three biological replicates performed for each group [24]. Egg formation in the uterus was observed under a confocal microscope (ZEISS, Germany).

### Statistical analysis

GraphPad Prism software (version 8.0) was used to perform statistical analyses using students's *t*-test or one way ANOVA. *P* ≤ 0.05 is considering as statistically significant.

## Results

### Dynamic transcript levels of GPCR*s* in differently development points of *S. japonicum*

**Fig. 1:**
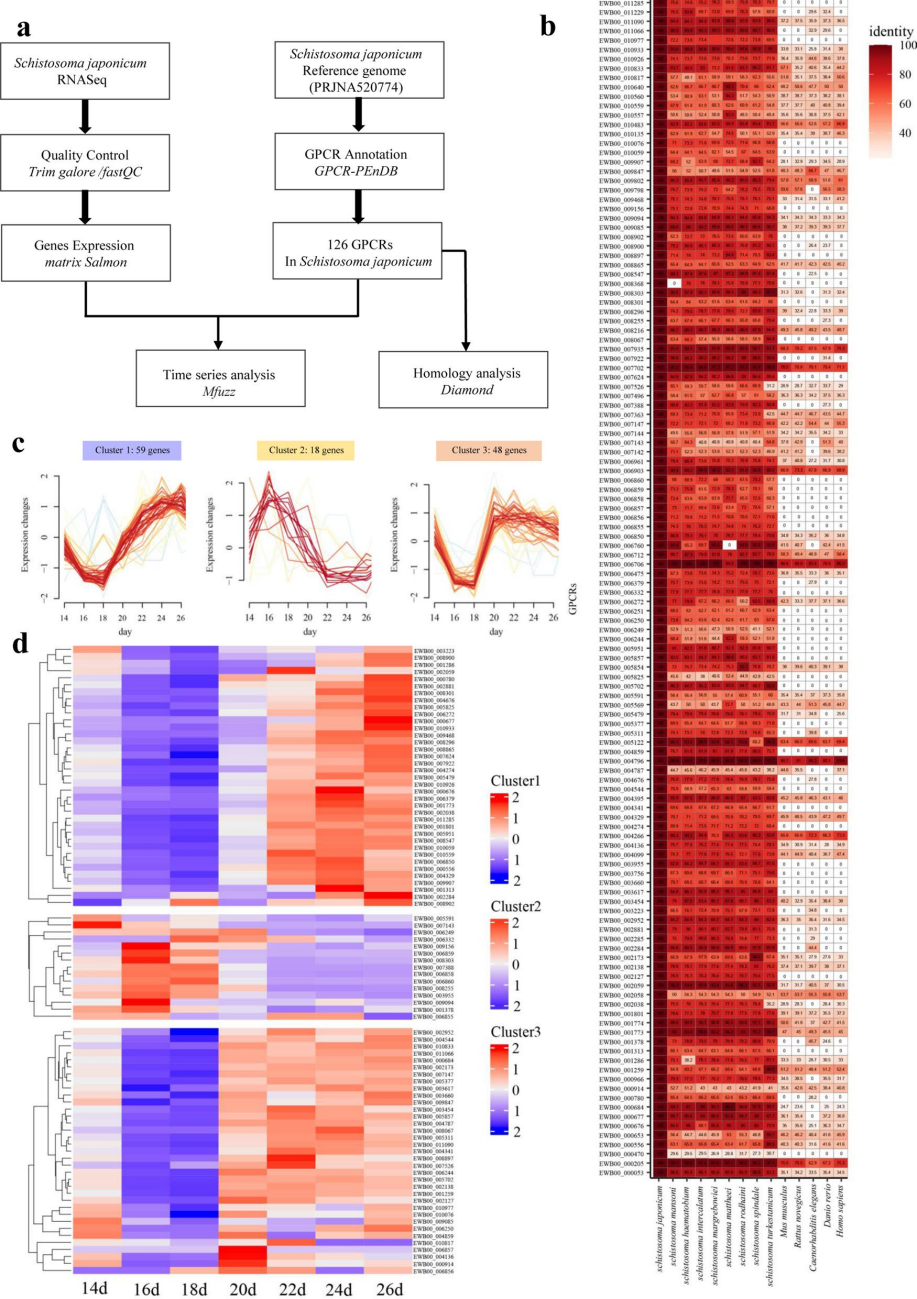
Bioinformatics analysis of GPCRs in *S. japonicum*. **a** Schematic representation of the bioinformatics approach employed to identify GPCR homologs in *S. japonicum*. **b** Comparative homology analysis of GPCRs in *S. japonicum* and other species. **c** Classification of transcript levels of *GPCRs* in *S. japonicum* across different development time points of adult development. **d** Heatmap showing the *GPCR* expressions at transcript levels in three clusters (cluster 1–3)

*S. japonicum* RNA seq datasets were bioinformatically analyzed, as shown in Fig. 1a. In total, 126 *GPCR* genes were identified and annotated in *S. japonicum* (Additional file 5: Table S2). The *S. japonicum* GPCRs were classified into five categories (Additional file 1: Figure S1a). With the highest number, 77 *GPCRs* belong to the rhodopsin and adrenergic-like receptors (Class A), followed by 5 to the heromone-like receptors (Class D), 3 to the Calcitonin and PTH-like receptors (Class B), 1 to the metabotropic-like receptors (Class C), and the remaining 40 GPCRs were unclassified (Additional file 1: Fig. S1a, Additional file 5: Table S2). In addition, the upstream ligands of these GPCRs were predicted, and the results showed that they could be classified into eight groups: amines, hormone proteins, lysosphingo lipid, nucleotide like, olfactory, peptide, rhodopsin, and unclassified ligands (Additional file 1: Fig. S1b, Additional file 5: Table S2). Interestingly, the two largest groups were putatively related to the amine and peptide classes. Sequence identity analysis indicated that two GPCR genes (EWB00_004796 and EWB00_006706) in *S. japonicum* exhibited a high degree of conservation with their homologous GPCRs in humans, mice, and rats, with over 80% amino acid sequence identity (Fig. 1b, Additional file 6: Table S3). Comparative analysis of GPCR homology between *S. japonicum* and *S. mansoni* revealed that 110 GPCRs in *S. japonicum* matched those in *S. mansoni*, with sequence similarities ranging from 32.7% to 96.3%. These matched GPCRs were categorized as follows: 89 in Class A, 5 in Class B, 3 in Class C, 6 in Class F (Frizzled), 5 others, and 2 unidentified (Additional file 2: Fig. S2a, Additional file 6: Table S3). Temporal expression profiling analyses indicated that these genes could be classified into three clusters (Fig. 1c and d). Cluster 1 consisted of 59 genes whose expression levels increased from day 18, around the time of male and female pairing, and continued to increase until day 26. Cluster 2, comprising 18 genes, showed a moderately consistent decrease in expression starting from day 16. Cluster 3 contained 48 genes whose expression levels also increased from day 18 and peaked around day 20. These dynamic transcript levels of *GPCR*s may reflect their potentially important functions for specific development points in *S. japonicum* (Fig. 1d).

### *Sj-Smo* and *Sj-imGPCR* were enriched in female* S. japonium*

**Fig. 2:**
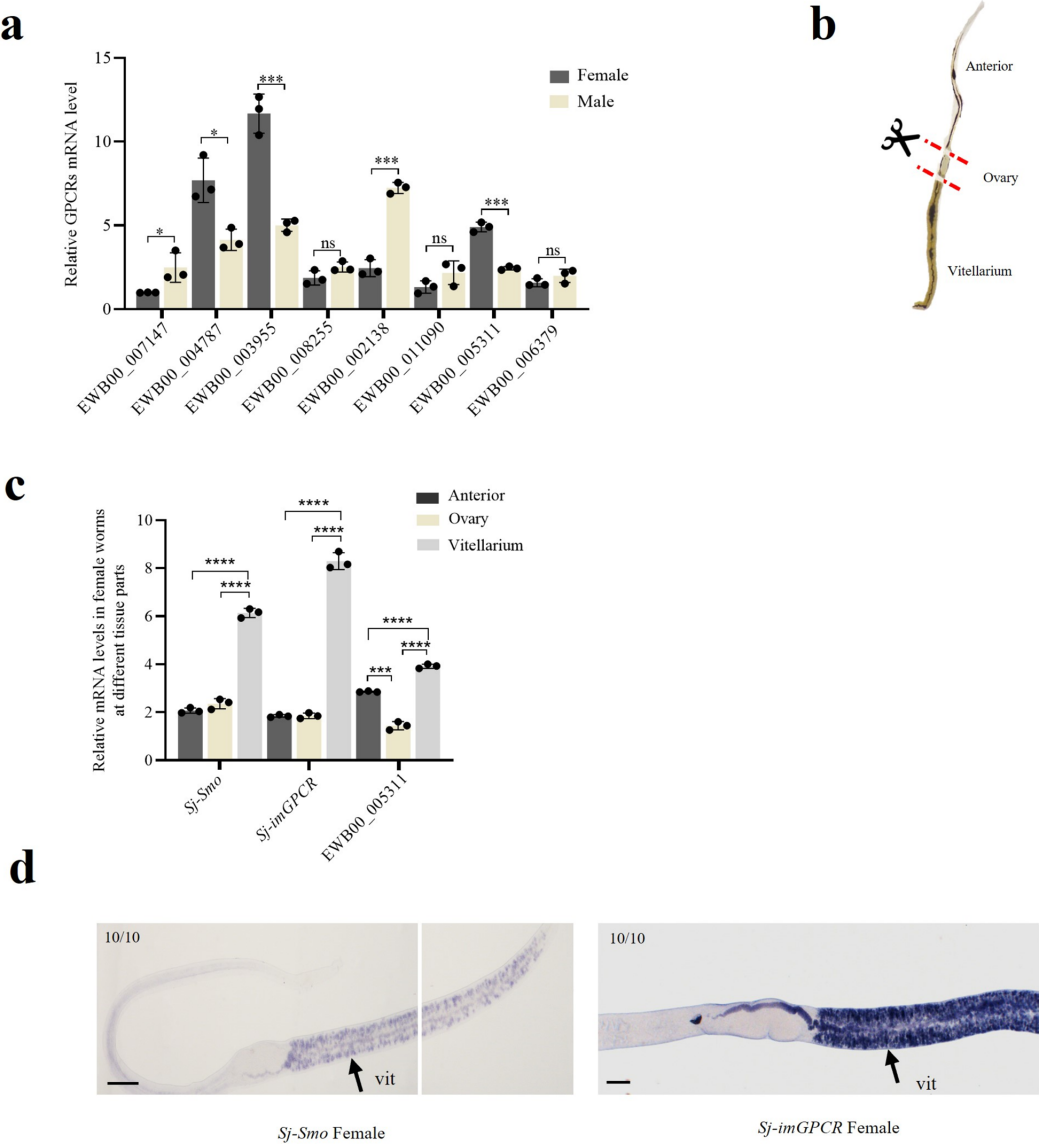
Analysis of *Sj-Smo* and *Sj-imGPCR* expressions at transcript levels using RT-qPCR and whole-mount in situ hybridization (WISH). **a** qPCR analysis of the expressions of *Sj-smo* and *Sj-imGPCR* at transcript levels between male and female. **b** Schematic representation of the dissection of adult female *S. japonicum.* **c** qPCR analysis of the expressions of *Sj-smo* (EWB00_004787), *Sj-imGPCR* (EWB00_003955), and EWB00_005311 in dissected parts of female *S. japonicum*. For panel a and c, data are represented as mean ± SEM of three independent experiments. ns indicates not significant, * *P* ≤ 0.05, ** *P* ≤ 0.01, *** *P* ≤ 0.001. **d** WISH analysis of the localization for *Sj-smo* and *Sj-imGPCR* in adult female *S. japonicum*. In each image, the number in the upper left corner indicates the number of females with similar staining. vit, vitellarium; ov, ovary. Numbers in corner indicate fraction of worms that are similar to those presented/total number of worms examined. Scale bar = 100 µm

Based on the *GPCR*s transcriptome analysis results, eight *GPCR*s were selected for subsequent studies. Firstly, primers were designed to amplify the full-length coding DNA sequences (CDS) of these eight *GPCR*s and it was confirmed that they were indeed transcribed in *S. japonicum* (Additional file 1: Figure S1c). The transcript levels between male and female adult *S. japonicum* were then evaluated using RT-qPCR. The primers for qPCR were evaluated for their amplification efficiency, and the results indicated that all the primer pairs exhibited efficiencies ranging from 95% to 105% (Additional File 4: Table S1). qPCR results indicated that EWB00_004787 (*Sj-Smo*), EWB00_003955 (*Sj-imGPCR*) and EWB00_005311 were highly expressed in female *S. japonicum* at the transcriptional level (Fig. 2a). To further determine the expression patterns of these *GPCR*s in females, female worms were dissected into three parts, including anterior, ovary, and vitellarium (Fig. 2b). RT-qPCR was used to evaluate their abundances in each part, and the results showed that three *GPCR*s, *Sj-Smo*, *Sj-imGPCR* and EWB00_005311, were significantly enriched in the vitellarium (Fig. 2c). To further corroborate these results, *Sj-Smo* and *Sj-imGPCR* were selected to perform WISH experiments and determine the localizations of *Sj-Smo* and *Sj-imGPCR* in adult females*.* These results further confirm their enrichment in the vitellarium of female worms (Fig. 2d).

### *Sj-Smo* and *Sj-imGPCR* colocalized with *Sj-DDR48*, which was significantly enriched in the vitellarium

**Fig. 3:**
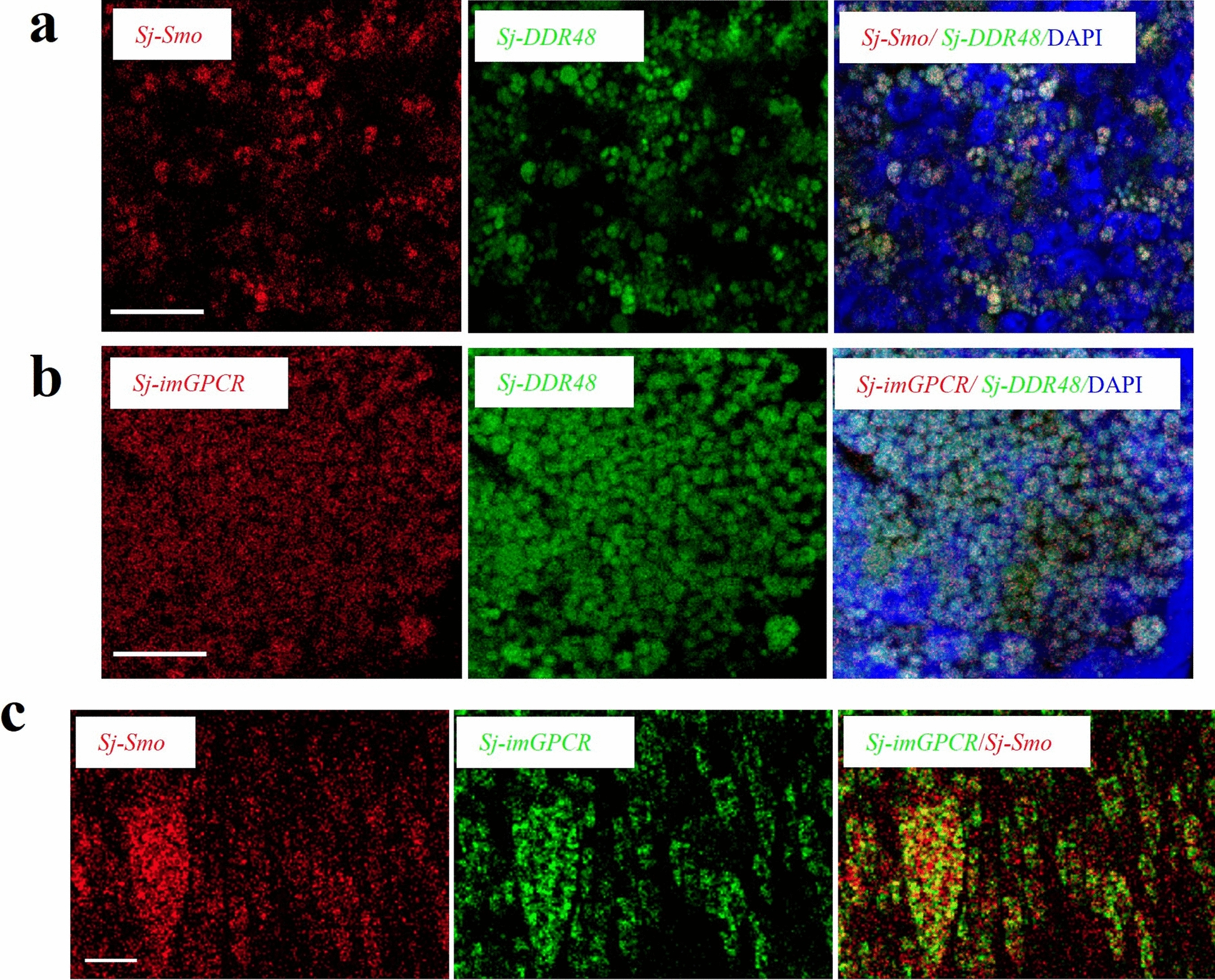
Double fluorescence in situ hybridization (FISH) analysis of *Sj-Smo* and *Sj-imGPCR* showed colocalization with the *Sj-DDR48*. **a** Double FISH analysis of *Sj-Smo* and *Sj-DDR48* revealed that *Sj*-Smo colocalized with *Sj*-*DDR48* within the vitellarium region of female worms. Cells were counterstained with DAPI (blue). DIG-labeled *Sj-Smo* is shown in red, and FITC-labeled *Sj-DDR48* is shown in green. Scale bar = 10 µm. **b** Double FISH analysis of *Sj-imGPCR* and *Sj-DDR48* in the female worm vitellarium. Cells were counterstained with DAPI (blue). DIG-labelled *Sj-imGPCR* is shown in red, and FITC-labeled *Sj-DDR48* is shown in green. Scale bar = 10 µm. **c** Double FISH analysis showing the colocalization of *Sj-Smo* and *Sj-imGPCR* in the vitellarium region of female *S. japonicum*. Abbreviations: vit, vitellarium; ov, ovary. FITC-labeled *Sj-imGPCR* is shown in green, and DIG-labeled *Sj-Smo* is shown in red. Scale bar = 10 µm

To corroborate these results, RNA FISH was used to further determine the localization of *Sj-Smo* and *Sj-imGPCR*. *Sj-DDR48* (EWB00_005782), a DNA damage-responsive protein 48, was selected. In our single-cell RNA sequencing study of *S. japonicum*, we found that *Sj-DDR48* was significantly enriched in the vitellarium of females and was selected as a control (Additional file 2: Fig. S2b). Upon performance of double FISH analyses, the results revealed that either *Sj-Smo* or *Sj-imGPCR* colocalized with *Sj-DDR48* (Fig. 3a–b). *Sj-Smo* and *Sj-imGPCR* exhibited pronounced colocalization within the vitellarium (Fig. 3c), suggesting functional convergent regulation of vitelline cell development in *S. japonicum*.

### Inhibition of *Sj-Smo* and *Sj-imGPCR* reduced cell proliferation in the vitellarium of adult females

**Fig. 4:**
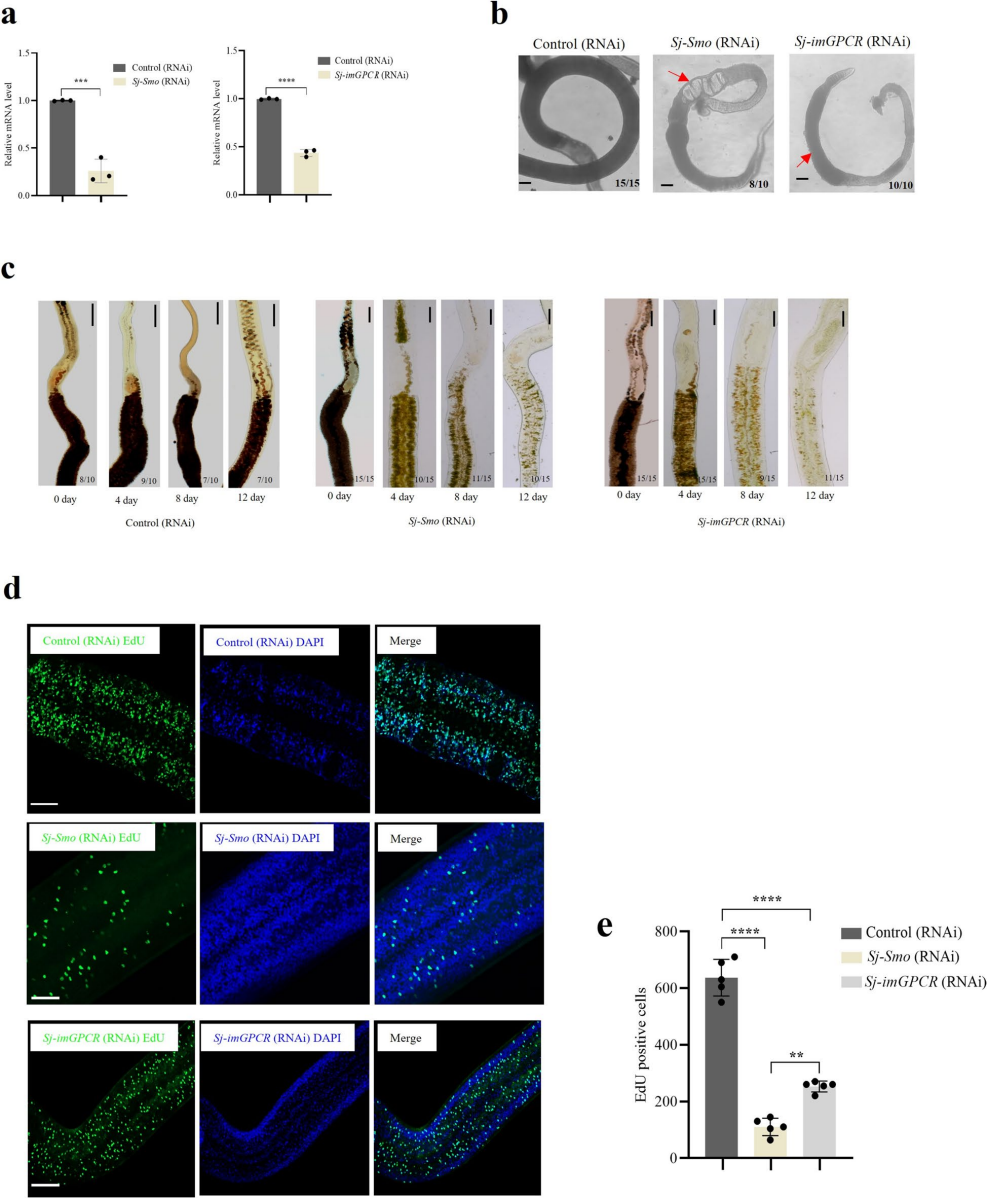
Inhibition of *Sj-Smo* and *Sj-imGPCR* led to reduced cell proliferation in the vitellarium of female worms. **a** qPCR analysis of *Sj-Smo* and *Sj-imGPCR* in females treated with *Sj-Smo* and *Sj-imGPCR* dsRNA. Data illustrate representative results with the mean and standard error derived (SEM) from triplicate experiments. *** *P* ≤ 0.001. **b** Morphological alterations of worms treated with *Sj-Smo* and *Sj-imGPCR* dsRNA. In each image, the number in the lower right corner indicates the number of female worms with similar conditions. Numbers in corner indicate fraction of worms similar to those presented/total number of worms examined. Scale bar = 100 μm. **c** RNAi against *Sj-Smo* and *Sj-imGPCR* led to vitellarium regression in female worms. Female schistosomes were stained with FastBlue BB on days 0, 4, 8, and 12 after dsRNA treatment, and stained worms were imaged using a microscope. Numbers in corner indicate fraction of worms that are similar to those presented/total number of worms examined. Scale = 100 μm. **d** EdU staining analysis of the vitellarium in female schistosomes treated with dsRNA *Sj-Smo* and *Sj-imGPCR*. Scale = 10 μm. The results show representative images from three biological replicates. **e** Quantitative analysis of EdU-positive cells in the vitellarium of female worms. After the female worms were treated with dsRNA, EdU labeling was performed and positive cells in captured areas of vitellarium were quantified using Image J. The data represent results with the mean ± standard error from three independent experiments, with five worms observed per experiment. **** *P* ≤ 0.0001

To determine the functional roles of *Sj-Smo* and *Sj-imGPCR,* RNA interference (RNAi) was performed using dsRNAs that specifically inhibited *Sj-Smo* and *Sj-imGPCR* at the transcriptional level. Upon the treatment of in vitro cultured adult worms with dsRNA, qPCR analyses of the treated worms indicated the transcript levels of *Sj-Smo* and *Sj-imGPCR* were significantly decreased, ranging from 57% to 81% (Fig. 4a). Interestingly, morphological alterations in the *Sj-Smo* and *Sj-imGPCR* inhibited parasites, for example, vacuolation was observed in the worm bodies (Fig. 4b). Next, the effects of *Sj-Smo* and *Sj-imGPCR* inhibition were determined on the development of the vitellarium by Fast Blue BB staining. The results showed reductions in vitelline droplets upon the suppression of *Sj-Smo* and *Sj-imGPCR* compared with the controls (Fig. 4c). Using the EdU incorporation assay, we observed decreased cell proliferation in the vitellarium of female worms following inhibition of *Sj-Smo* and *Sj-imGPCR* (Fig. 4d). Quantitative analysis of the EdU-positive cells with EdU staining in the vitellarium was conducted using ImageJ, and the results showed 73% and 54% reductions in cell proliferation following inhibition of *Sj-Smo* and *Sj-imGPCR*, respectively (Fig. 4e).

### Inhibition of* Sj-Smo* and *Sj-imGPCR* decreased egg production in adult females

**Fig. 5:**
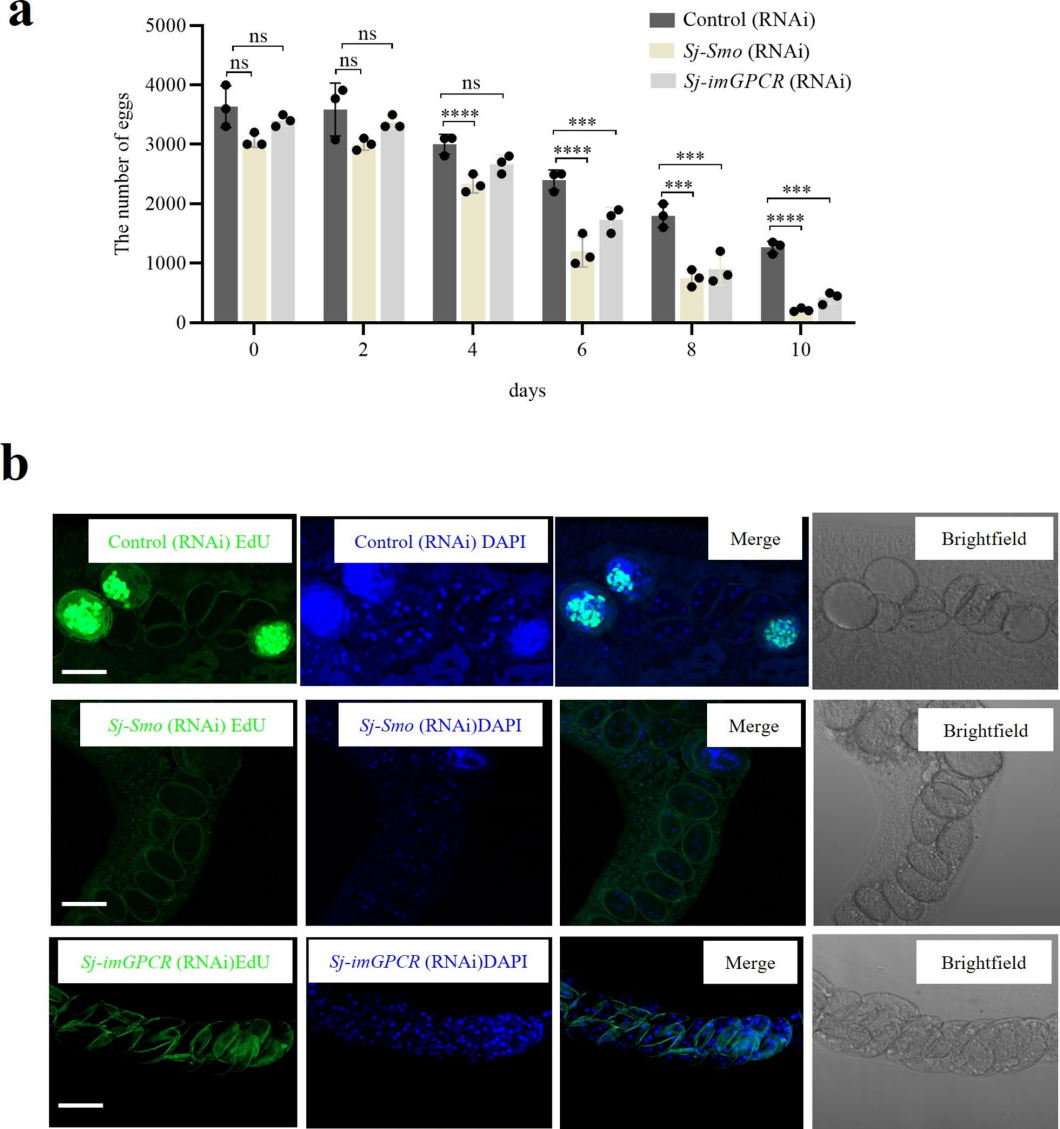
*Sj-Smo* and *Sj-imGPCR* inhibition decreased the number of egg produced in treated females. **a** *Sj-Smo* and *Sj-imGPCR* inhibition decreased the egg production in treated females. Data illustrate representative results as mean ± standard error derived from the experiments of three wells. ns means not significant, *** *P* ≤ 0.001, **** *P* ≤ 0.0001. **b** Estimation of cell proliferation in eggs from oviduct of dsRNA-treated females by EdU staining. Female worms were treated with dsRNA and then eggs in oviduct were observed at 12 d posttreatment. Scale bar = 10 μm

We also monitored egg production in *Sj-Smo* and *Sj-imGPCR* inhibited females during the treatments. The results indicated that inhibition of both *Sj-Smo* and *Sj-imGPCR* resulted in a decreased number of eggs produced, with a significant reduction observed starting from 6 days posttreatment compared with the control group (Fig. 5a). Compared with the control group, we noted that nearly all eggs observed in the uterus of RNAi-treated worms were negative for EdU staining (Fig. 5b), implying that *Sj-Smo* and *Sj-imGPCR* inhibition may influence cell proliferation in eggs.

## Discussion

GPCRs play crucial roles in cellular signal transduction, converting extracellular signals, such as light, odors, hormones, and neurotransmitters, into intracellular signals that trigger a series of biological responses [25, 26]. In the malaria parasite, PfSR25 was identified as a sensor for K + shifts, affecting the growth and development of the parasite by GPCR [27]. In addition, GPCRs play a central role in insect physiology and toxicology by participating in the regulation of reproduction, growth, development, stress responses, feeding behaviors, and other physiological processes [28]. In the present study, we systematically analyzed GPCRs in the *S. japonicum* transcriptome across different developmental stages and identified 126 GPCR genes. The most abundant class in *S. japonicum* is class A rhodopsin, which is also the largest GPCR family and is primarily a receptor for hormones and neurotransmitters. Comparative analysis with *S. mansoni* GPCRs revealed 25 homologs with > 80% sequence similarity, including 21 class A, 2 class B, 1 class C, and 1 unclassified receptor [8, 29]. Among these, the *S. japonicum* dopamine D2-like receptor (*D2R*) gene showed the highest sequence homology (96.3%) with the *S. mansoni* ortholog. Studies have shown that in fruit flies, the D2-like receptor functions in the glial cells of the blood–brain barrier (BBB) and regulates complex courtship behaviors [30]. Based on the abundance and homology analysis, eight highly expressed GPCRs were selected. The full-length CDS of these GPCRs was amplified and sequenced to confirm their presence in *S. japonicum*. Subsequently, qPCR was performed to determine the abundance of these genes. On the basis of these results, two genes were identified in the vitellarium, *Sj-Smo* and *Sj-imGPCR*. Next, dsRNA was designed and synthesized to inhibit the expression of these two GPCRs. RNAi of *Sj-Smo* and *Sj-imGPCR* significantly impaired the growth and development of the worm, with degeneration of the vitellarium, decreased egg production, reduced egg viability, damage to the worm’s surface, and affected cell proliferation (Fig. 4b–d). These results indicate that *Sj-Smo* and *Sj-imGPCR* may regulate egg production in *S. japonicum*. Further analysis identified the *S. mansoni* homolog of *Sj-Smo* as a frizzled domain-containing protein (Smp_125160) with 44.7% amino acid identity, which primarily functions through the Wnt signaling pathway and is associated with parasite development, reproduction, and tissue homeostasis [31]. Previous studies have shown that schistosome-frizzled proteins (frizzled-5/7/9) can be targeted by female-enriched miRNAs (Bantam and miR-1989), with frizzled suppression leading to decreased egg production and ovarian structural alterations, underscoring their critical role in ovarian development and egg maturation [32]. The homologous gene of *Sj-imGPCR* was identified as a Rhopsn4 domain-containing protein (Smp_049330) with 82.8% sequence identity. This receptor is hypothesized to mediate olfactory signaling in the miracidial stage of *S. mansoni*, aiding host recognition and localization of intermediate freshwater snail hosts [8]. Currently, understanding of Rhopsn4 protein function in schistosomes remains limited, and no homologs have been identified in *C. elegans* or planarians.

To determine GPCR expression in different anatomical regions of *S. japonicum*, adult female worms were divided into three anatomical regions. Owing to limitations in fine dissection techniques, each part has mixed tissues, for example, the anterior region contains oral and ventral suckers and uterus, and the vitellarium includes intestinal tract structures. Consequently, the relative gene expressions across segments may reflect mixed tissue contributions. WISH analyses indicated that the investigated *GPCRs* were significantly localized in the vitellarium. Subsequent studies should employ laser microdissection techniques to further enhance the precision of tissue separation. Analysis of the protein–protein interaction network for *Sj-Smo* suggested its potential role in the Hedgehog (Hh) signaling pathway (Additional file 3: Fig. S3b) [33]. The Hh signaling pathway is a highly conserved evolutionary pathway that is crucial for normal embryonic development and the maintenance, renewal, and regeneration of adult tissues (Additional file 3: Fig. S3c and d) [34]. In mammals, canonical Hh signaling involves Hh ligand binding to the Patched (PTCH) receptor, a transmembrane protein that constitutively inhibits *Smo* activity in the absence of ligands, thereby regulating the activation of this GPCR-like protein [35]. Activation of *Smo* further promotes downstream signal transduction, ultimately leading to the activation of the transcription factor *gli* (Glioma-associated oncogene homolog), a family of zinc-finger proteins (Gli1, Gli2, Gli3) that regulate Hh target genes [35]. In the absence of Hh signals, Gli is phosphorylated and acts as a transcriptional repressor [36, 37]. Protein–protein interaction analysis also indicated that *Sj-Smo* is potentially involved in core Hh pathway components (ptc and gli1) and secondary interactors, including dispatched (a lipid-modified Hh transporter critical for long-range signaling and tissue patterning) [38]. This dispatched function is essential for the long-range signaling of Hh proteins, enabling these signaling molecules to diffuse from source cells and regulate tissue patterning during development [39]. Sufu serves as a crucial negative regulator of the pathway by interacting with *gli* transcription factors to control their activity and suppress the Hh signaling pathway [40]. Sufu binds directly to Gli proteins, preventing their translocation to the nucleus and maintaining a balance between activated and repressed forms of Gli [39]. This regulatory mechanism precisely controls Hh signaling and prevents pathological activation. RNAi-mediated *Sj-Smo* inhibition disrupts Hh pathway functionality in schistosomes, causing dysregulation of *gli* and *ptc* expression.

## Conclusions

We systematically analyzed GPCR homologs in *S. japonicum* transcriptomes and identified 126 *GPCR*s. Among these, *Sj-Smo* and *Sj-imGPCR* showed significant enrichment in the vitellarium of female worms. Using RNA interference, we found that *Sj-Smo* and *Sj-imGPCR* might be involved in egg production by regulating the functions of the vitellarium in female *S. japonicum*.

## Supplementary information

Additional file 1: Figure S1. *S. japonicum* *GPCR* classification and predicted upstream ligands. (**a**) Heatmap showing the transcript levels of *GPCRs* across different development stages. Class A included rhodopsin and adrenergic-like receptors (77 *GPCR*s), Class B included calcitonin and PTH-like receptors (3 *GPCR*s), Class C included Metabotropic-like receptors (1 *GPCR*), Class D included hormone-like receptors (5 *GPCR*s), and the unclassified (40 *GPCR*s) category included *GPCR*s with uncertain classification information. (**b**) Heatmap showing the transcript levels of predicted upstream ligands of *GPCR*s across different developmental points. They were categorized into different groups: amines (8 ligands), hormone proteins (1 ligands), lysosphingo lipids (4 ligands), nucleotide-like (2 ligands), olfactory (4 ligands), peptides (56 ligands), rhodopsin (2 ligands), and unclassified ligands (49 ligands). (**c)** Agarose gel electrophoresis analysis of PCR products to amplify the full-length CDS of eight selected *GPCR*s: EWB00_005311, EWB00_003955, EWB00_002138, EWB00_004787, EWB00_008255, EWB00_006379, EWB00_0011090 and EWB00_007147. M, DNA Marker.

Additional file 2: Figure S2. Homology analysis of *GPCRs* in *S. japonicum* and *S. mansoni* and the *Sj-DDR48* WISH. (**a**) The numbers in the heatmap indicate identity values. Among them, class A, rhodopsin and adrenergic-like receptors; class B, PTH-like receptors; class C, metabotropic-like receptors; class F, frizzled. (**b**) WISH analysis of the localization for *Sj-DDR48* in adult female *S. japonicum*. In each image, the number in the upper left corner indicates the number of females with similar patterns. Scale bar = 100 µm.

Additional file 3: Figure S3. *Sj-Smo GPCR* may function in the Hh signaling pathway. (**a**) KEGG enrichment analysis was conducted for eight GPCR genes, including *Sj-Smo* and *Sj-imGPCR*. (**b**) STRING analysis was performed to examine protein–protein interactions by inputting *Sj-Smo* and Hh pathway genes associated with the Hh pathway. qPCR analysis was used to assess the expression levels of *gli* (**c**) and *ptc* (**d**), which may be involved in the Hh pathway following *Sj-Smo* inhibition in *S. japonicum*. The data are representative of mean ± standard error from three independent experiments. *** *P* ≤ 0.001.

Additional file 4: Table S1. List of primers used in the present study.

Additional file 5: Table S2. Original data table for *GPCR* classification and ligand prediction in *S. japonicum*. In the table, the first column is the *s.j GPCR* genes ID, the second column is the *GPCR*s amino acid length, the third column is the class predicted by the GPCRPred database, the fourth column is the upstream ligand of *GPCR*s predicted by the GPCRPred database, and the last column is the amino acid sequence of the gene.

Additional file 6: Table S3. Analysis of *GPCR* genes homology between *S. japonicum* and other species. The first column is the *s.j GPCR* genes ID, the second column is the homologous *GPCR* genes ID in the corresponding species, the third column is the homology percentage, the fourth column is the expectation value (E-value), and the last column is the similarity strength value (bitscore). The notation "N/A" in the table indicates that no homologous *GPCR* was found in a different species.

## Data Availability

Data supporting the main conclusions of this study are included in the manuscript.
