# Supplementary figures and images for: Genome-wide identification and functional characterization of GPCR family genes reveal their key roles in the vitellarium development and egg production in Schistosoma japonicum
Source: Parasit Vectors. 2025 Jul 17;18:286. doi: 10.1186/s13071-025-06929-2 (PMC12273374; doi:10.1186/s13071-025-06929-2)

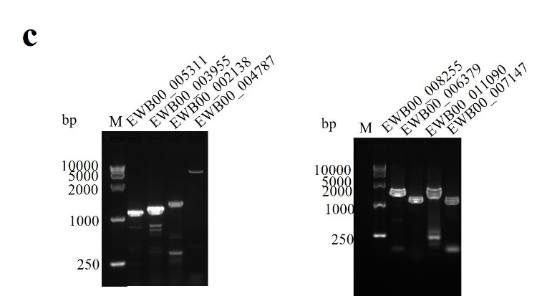

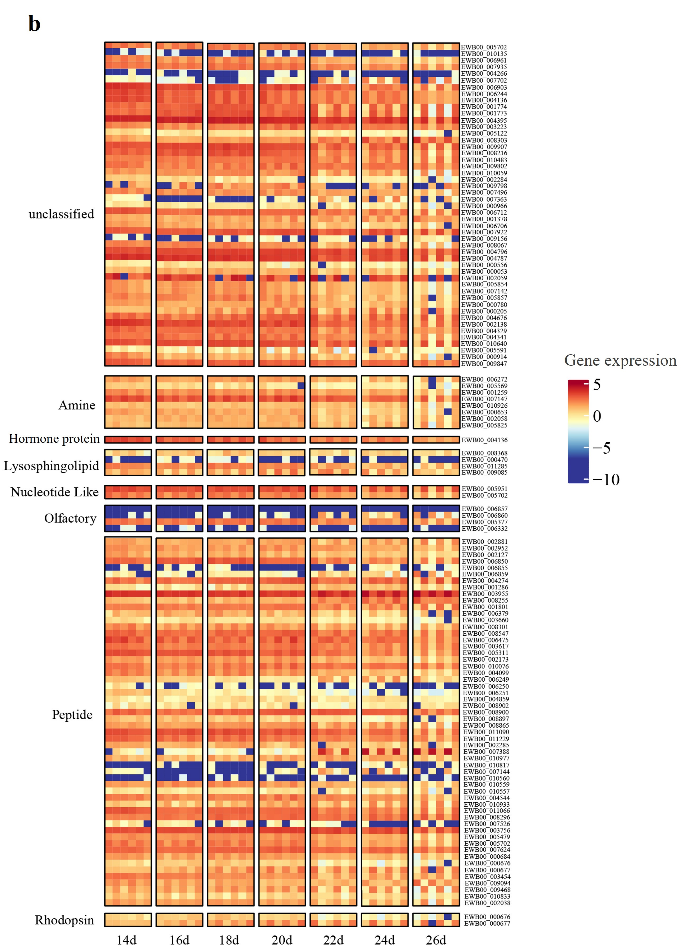

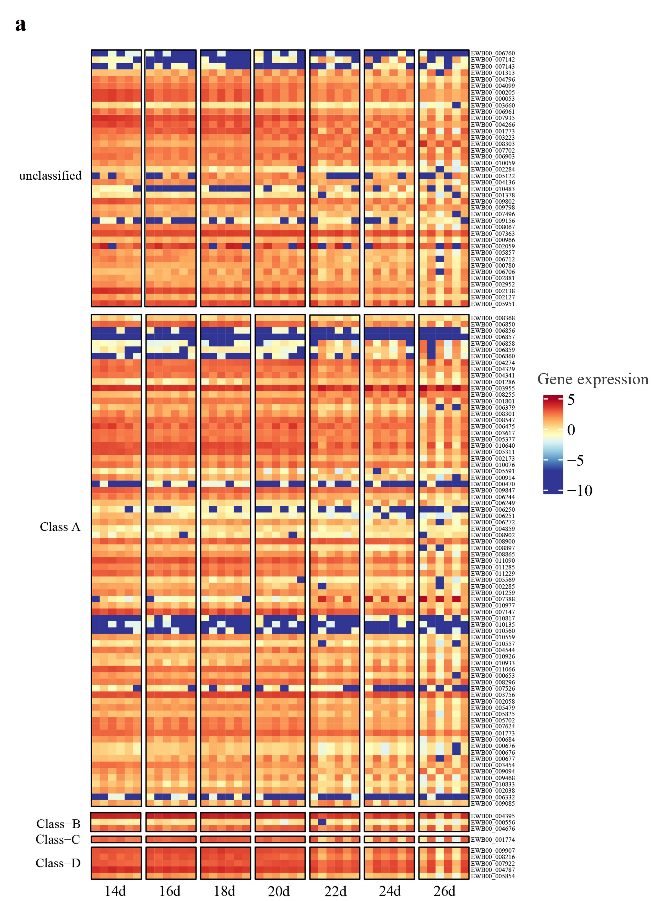

Supplement: Supplementary file 1 — Additional file 1: Figure S1. S. japonicum GPCR classification and predicted upstream ligands. (a) Heatmap showing the transcript levels of GPCRs across different development stages. Class A included rhodopsin and adrenergic-like receptors (77 GPCRs), Class B included calcitonin and PTH-like receptors (3 GPCRs), Class C included Metabotropic-like receptors (1 GPCR), Class D included hormone-like receptors (5 GPCRs), and the unclassified (40 GPCRs) category included GPCRs with uncertain classification information. (b) Heatmap showing the transcript levels of predicted upstream ligands of GPCRs across different developmental points. They were categorized into different groups: amines (8 ligands), hormone proteins (1 ligands), lysosphingo lipids (4 ligands), nucleotide-like (2 ligands), olfactory (4 ligands), peptides (56 ligands), rhodopsin (2 ligands), and unclassified ligands (49 ligands). (c) Agarose gel electrophoresis analysis of PCR products to amplify the full-length CDS of eight selected GPCRs: EWB00_005311, EWB00_003955, EWB00_002138, EWB00_004787, EWB00_008255, EWB00_006379, EWB00_0011090 and EWB00_007147. M, DNA Marker. [file 13071_2025_6929_MOESM1_ESM.docx]

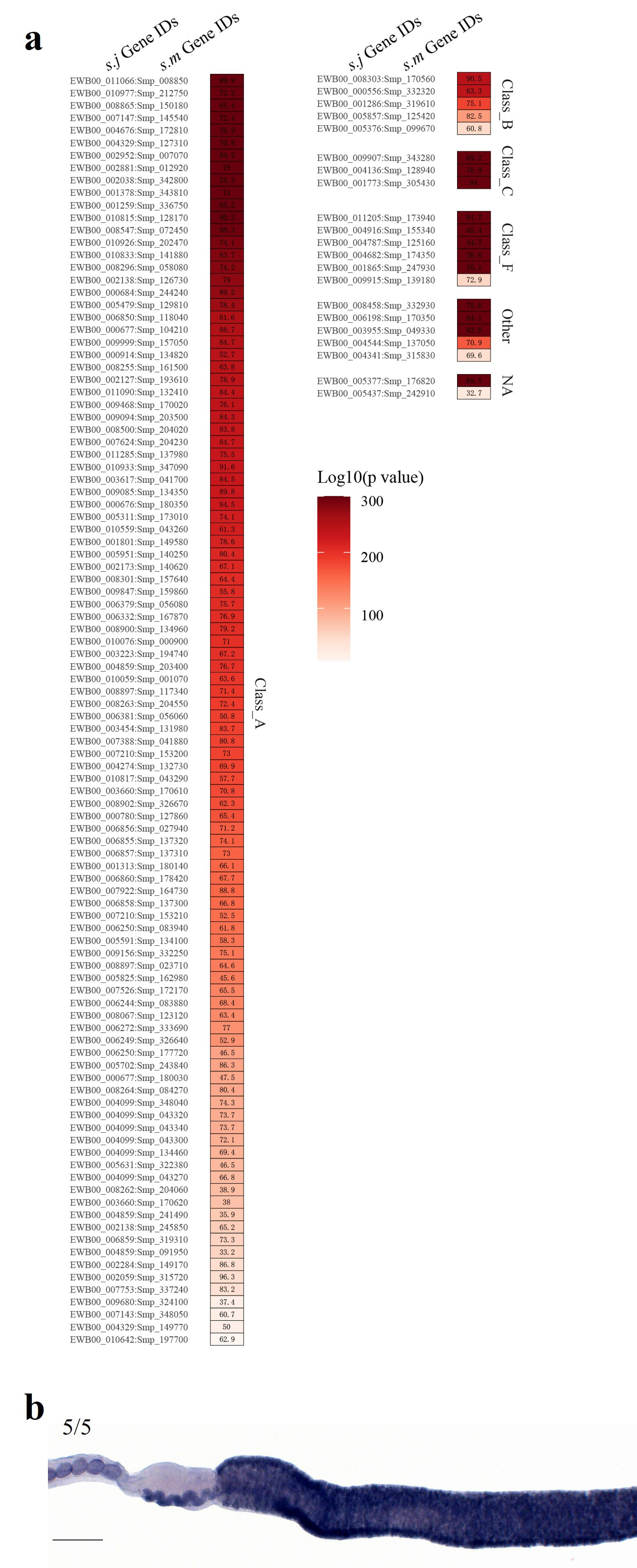

Supplement: Supplementary file 2 — Additional file 2: Figure S2. Homology analysis of GPCRs in S. japonicum and S. mansoni and the Sj-DDR48 WISH. (a) The numbers in the heatmap indicate identity values. Among them, class A, rhodopsin and adrenergic-like receptors; class B, PTH-like receptors; class C, metabotropic-like receptors; class F, frizzled. (b) WISH analysis of the localization for Sj-DDR48 in adult female S. japonicum. In each image, the number in the upper left corner indicates the number of females with similar patterns. Scale bar = 100 µm. [file 13071_2025_6929_MOESM2_ESM.tif]

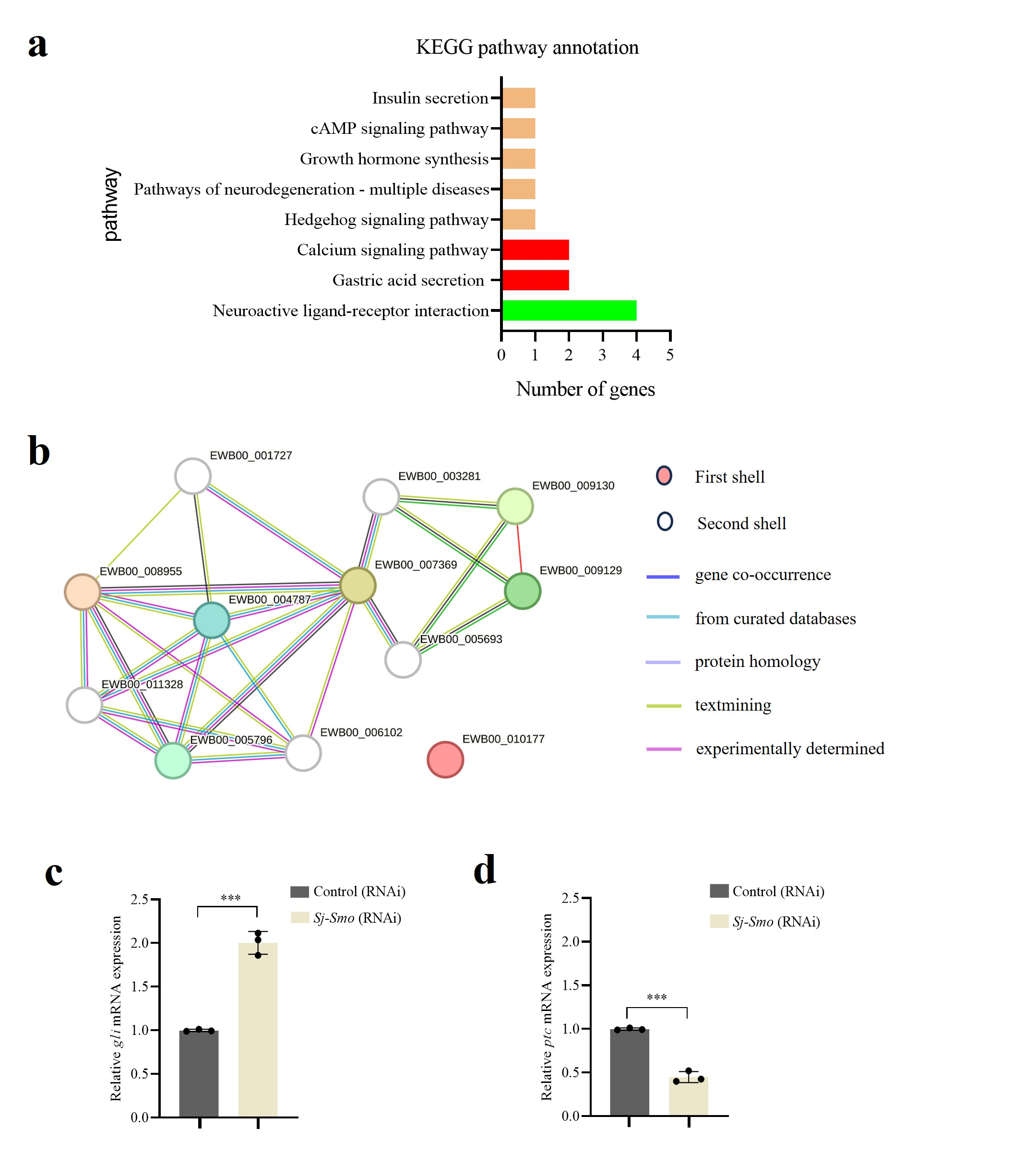

Supplement: Supplementary file 3 — Additional file 3: Figure S3. Sj-Smo GPCR may function in the Hh signaling pathway. (a) KEGG enrichment analysis was conducted for eight GPCR genes, including Sj-Smo and Sj-imGPCR. (b) STRING analysis was performed to examine protein–protein interactions by inputting Sj-Smo and Hh pathway genes associated with the Hh pathway. qPCR analysis was used to assess the expression levels of gli (c) and ptc (d), which may be involved in the Hh pathway following Sj-Smo inhibition in S. japonicum. The data are representative of mean ± standard error from three independent experiments. *** P ≤ 0.001. [file 13071_2025_6929_MOESM3_ESM.tif]
